# Biochar one-off application for paddy soil ^15^N loss improvement: evidence from a two-year experiment

**DOI:** 10.3389/fpls.2025.1683435

**Published:** 2025-10-28

**Authors:** Jiping Gao, Yanghui Sui, Zhongcheng Zhang, Kai Zhang, Hongfang Jiang, Yuzhuo Liu, Zhongcheng Sun, Xinyue Bing, Yanze Zhao, Wenzhong Zhang

**Affiliations:** ^1^ Rice Research Institute, National and Local Joint Engineering Laboratory of Japonica Rice Breeding and Cultivation Technology in North China, Shenyang Agricultural University, Shenyang, China; ^2^ Corn Research Institute, Liaoning Academy of Agricultural Sciences, Shenyang, China

**Keywords:** biochar, N fixation, rice growth stages, ^15^N fertilizer fate, staged fertilization

## Abstract

The aim of this study was to quantify the impact of biochar one-off application on split application of nitrogen fertilizers. We used the ^15^N tracer technique to explore the effects of biochar on greenhouse gas (GHG) emissions and NUE during three growth stages (tillering, panicle initiation, and ripening). Total nine treatments incorporated three biochar levels (0, C0; 15, C1; 45 t ha^−1^, C2) with three N levels (0, N0; 168, N1; conventional N fertilization at 210 kg N ha^−1^, N2). The high N_2_O emission rate during the tillering stage was significantly affected by biochar application and its interaction with N fertilization in both years, with 2016 yielding higher emissions (15.8%–65.2% of the total). Optimizing biochar application with a focus on the tillering and panicle initiation stages can helped mitigate global warming potential (GWP) in the initial application. Rice yields were highest in N2C0 and N2C2 treatments across years, which were 13.0% and 8.5% higher than yield in N1C0, respectively. The base fertilizers in 2 years reduced the ^15^N loss rate (NLR) in N1C2 treatment by 49.5% and 38.6% compared with N1C0, respectively. In the first year, the N recovery efficiency (NRE) in N1C2 treatment decreased by 55.2%, 44.0%, and 21.4% for base, tiller, and panicle fertilizers, respectively, compared to and N2C1 decreased the NRE of the base fertilizers by 27.9% in the following year. No significant differences in the NRE of tiller and panicle fertilizers were observed between N1C1 and N2C1 treatments in the following year. This study underscores the potential of biochar as a an environmentally friendly soil amendment for N loss reduction in rice systems. Biochar one-off application plays a role in mitigating GHG emissions, particularly during different fertilization periods which contributes to more sustainable agricultural practices.

## 1 Introduction

Rice is a primary staple food, with approximately 50% of the world’s population depending on it for calories and mineral nutrient intake (Xiao et al., 2025; Ren et al., 2022). Nitrogen (N) is an essential macronutrient that largely determines crop yield during agricultural production (Xu et al., 2012). However, excessive N fertilization has resulted in low nitrogen use efficiency (NUE) and can cause agricultural non-point source pollution (Sun et al., 2018; Zhang et al., 2019a). Worldwide, agricultural production contributes to approximately 67 Tg of nitrogen lost annually, representing 45% of the total N output (Liu et al., 2010). Surface runoff and nitrous oxide (N_2_O) are major pathways for agricultural N losses (Shi et al., 2025; Cherobim et al., 2017), which can be exacerbated by climate change (Eekhout et al., 2018). Based on this background, climate-smart agriculture has appeared to be a strategic solution aimed at achieving food security, addressing climate challenges, enhancing agricultural productivity, and increasing agricultural sustainability (Bhatnagar et al., 2024). In recent years, synergies of soil organic carbon and nitrogen in agricultural production have attracted much attention, that is, simultaneously achieving soil carbon sequestration and nitrogen reduction through the same technology (Wang et al., 2025). Biochar is regarded as a potential excellent carrier for achieving this goal (Lehmann et al., 2021).

Biochar, a stable carbon substance with a large surface area produced via pyrolysis of agricultural waste under anaerobic or oxygen-limited conditions, is gaining importance day by day in agriculture and has many benefits in improving soil fertility and yield (Chen et al., 2022; Dong et al., 2020), mitigating climate change (Amirahmadi et al., 2024; Song et al., 2019), and rehabilitating contaminated farmland (Zhang et al., 2025a; Yang et al., 2022a; Nie et al., 2021). Notably, biochar combined with N fertilizer has certain advantages for reducing N loss and improving NUE (Jin et al., 2024; Zhao et al., 2024; Gao et al., 2022). Jia et al. (2021) showed a 20% increase in NUE under biochar combined with N fertilizer compared with N fertilizer alone. Nevertheless, the effect of biochar one-off application on NUE in the different fertilization stages for rice remains unclear.

Meanwhile, biochar exhibits biochemical recalcitrance due to its highly porous structure and resistance to chemical treatment (Zimmerman et al., 2011), and its incorporation into fertile soils has produced varied effects on N_2_O emissions. Previous studies have reported that biochar had no significant effect on N_2_O emissions (Wu et al., 2019), but other studies suggested that it had a facilitation effect (Yoo et al., 2018). Moreover, biochar combined with N fertilizer has also been shown to lower N_2_O loss from soil (Puga et al., 2020), possibly due to a decreased population of denitrifiers in soils under biochar treatment compared to only N treatment (Liao et al., 2020). The underlying mechanisms are ascribed to high-dose biochar that reduced the abundance of *Sphingomonas* by reducing complex aromatic carbon compounds that this genus utilizes, leading to an increase in soil total nitrogen (TN) and a decrease in N_2_O at the end of the early rice season (Yang et al., 2022b). To better explain the effect of biochar on soil GHG emissions, field-aged biochar mitigates soil CO_2_ emissions and enhances short-term C sequestration primarily by reducing the bioavailability of its dissolved organic matter (DOM) and promoting its humification (Pan et al., 2024). Some studies have suggested that biochar may reduce cumulative N_2_O emissions by modifying nitrogen availability (Pokharel et al., 2018; Wu et al., 2020) and soil aeration (Hagemann et al., 2017), as well as by influencing nitrite-oxidizing bacteria (NOB) (Yu et al., 2024) and enhancing nutrient retention (El-Naggar et al., 2019). However, other studies have reported that the addition of biochar to Black Chernozem did not significantly affect N_2_O emissions in the short term (Cheng et al., 2012). Low-dose biochar enhances NUE in crops by minimizing N loss; particularly, high pyrolyzed biochar tends to increase ammonium and reduce phosphorus in 2 years (Zhao et al., 2023).

Previous research demonstrated that the split application of nitrogen fertilizers enhances nitrogen use efficiency by aligning the applied nitrogen with crop needs (e.g., when acquired during early tillering stages to produce a high number of panicles and a high percentage of filled spikelets) (Zhou, 2019). Nevertheless, research on the impact of biochar on the effectiveness of base, tiller, and panicle fertilizers in rice cultivation is rare. We aimed to find a method to achieve a more sustainable and stable increase in N recovery efficiency and reduce GHG emissions at rice key stages. The impact of biochar amendment on N_2_O emissions during the tillering stage, jointing–booting stage, and blooming and fruiting periods in paddy soils remains unknown. Multiple studies have employed ^15^N-labeled fertilizer to trace ^15^N in soil, thereby assessing the recovery and fate of applied nitrogen in planting systems (Du et al., 2022; Quan et al., 2020). To date, very little literature is available regarding the effectiveness of one-off biochar on the N recovery efficiency of growth stage-specific fertilizer and the quantified N_2_O emission rate of different stages (tillering, panicle, and ripening). We hypothesized that i) biochar one-off amendment would decrease N_2_O emissions in the tillering stage more than in the jointing–booting stage and blooming and fruiting periods in paddy soils and ii) biochar one-off amendment in pots would decrease the N recovery efficiency (NRE) of base fertilizers but increase soil residual N. Therefore, this study used ^15^N-labeled fertilizer combined with biochar as a slow-release nitrogen fertilizer to assess the N recovery efficiency of base, tiller, and panicle fertilizers while monitoring the dynamics of ^15^N in the soil and gaseous nitrogen losses from the fertilizer.

## 2 Materials and methods

### 2.1 Experimental site

**Figure 1:**
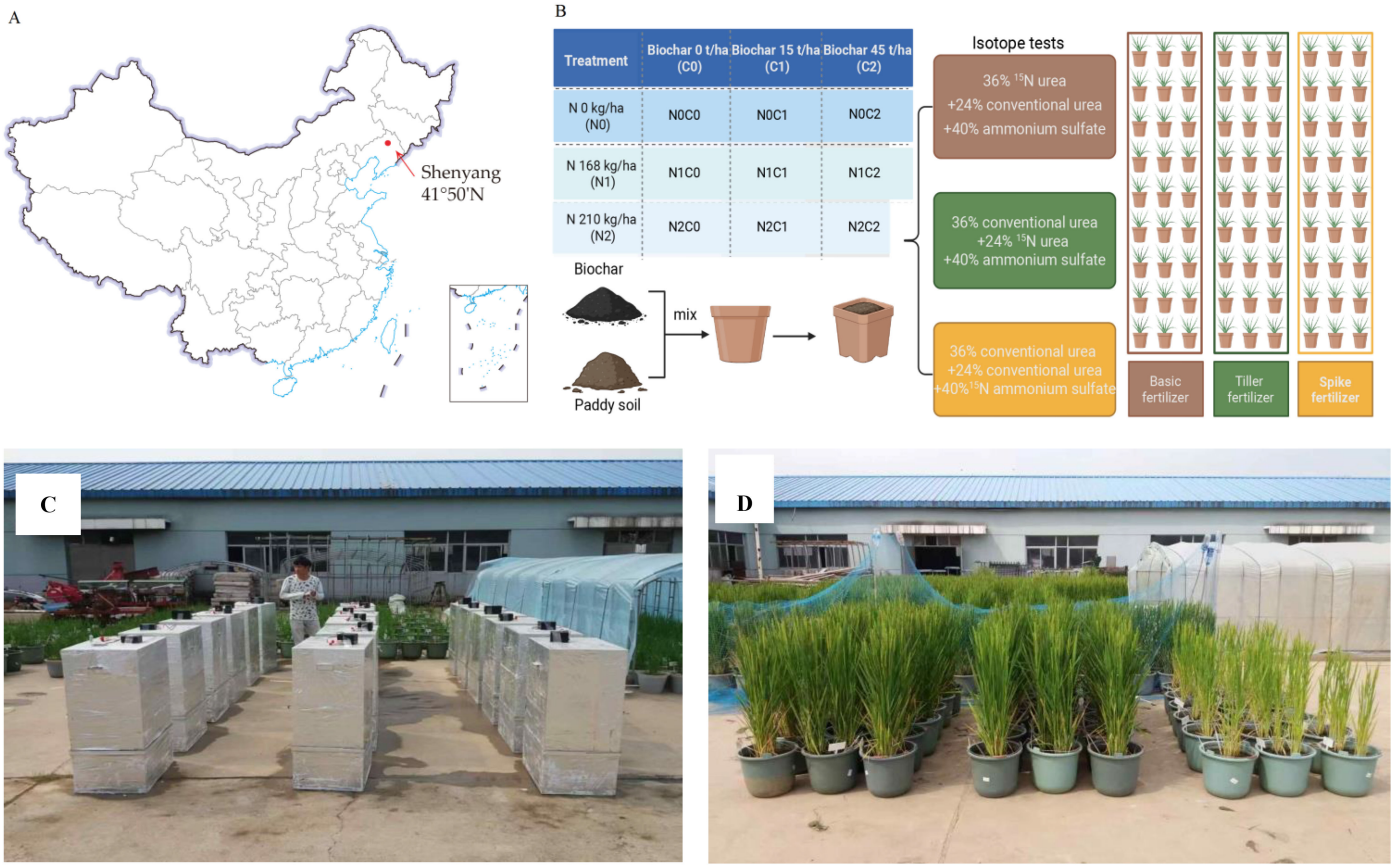
The experimental site and design of the study. **(A)** Location of experimental site (marked by red arrow) and **(B)** Schematic representation of the experimental design for nitrogen uses at various rice growth stages. During the growth period rice received base fertilizer, tillering fertilizer, and panicle fertilizer. The ^15^N isotope test was used for the nitrogen fertilizer utilization test. **(C)** Gas collection site and **(D)** Pot experiment.

The experimental site was conducted during two rice growing seasons (2016−2017) at a pot experiment field in the Rice Institute, Shenyang Agricultural University (41°50′N, 123°24′E), China (**Figure 1A**). This area has a typical semi-humid temperate continental monsoon climate with mean annual air temperatures of 8.3°C and 9.7°C and total precipitation of 500 and 423 mm, in 2016 and 2017, respectively. The soil was classified as Histosols with a bulk density of 1.46 g cm^−3^ (Gao et al., 2019).

### 2.2 Experimental design

Each pot was filled with 14 kg of dried soil. Biochar was derived from maize straw with a maximum pyrolysis temperature of 400 °C. Biochar was applied in 2016. A similar dose of biochar was applied to the soil surface and then manually mixed. More detailed properties of the basic soil and biochar are listed in **Table 1**.

**Table 1:** The fundamental characteristics of the unamended soil and maize straw biochar.

	Unamended soil	Biochar
pH	6.8	9.8
Total C (g kg^−1^)	15.39	624.2
Total N (g kg^−1^)	1.87	20.0
Total P (g kg^−1^)	0.66	1.19
Available N (mg kg^−1^)	73.6	40.68
Available P (mg kg^−1^)	16.5	32.3
Available K (mg kg^−1^)	155.7	100.83
Ash content (%)	/	14.8
Superficial area (m^2^ g^−1^)	/	34.9
Mean aperture dimension (nm)	/	43.2

Rice (cv. Shennong 265, *Oryza sativa* L. ssp. *japonica*) was planted in this study, which is widely cultivated locally. The fertilizer doses in this study were 0 kg N ha^−1^ (N0), 168 kg N ha^−1^ (N1), and 210 kg N ha^−1^ (N2), and the three biochar application rates were 0%, 0.7%, and 2.1%, w/w, equivalent to 0, 15, and 45 t ha^−1^ (C0, C1, and C2), respectively. In this experiment, the ^15^N-labeled fertilizer was applied to different pots (27 pots/period) in three periods, in order to investigate N utilization in different fertilization periods. The specific steps are detailed in **Figure 1**. In this experiment, 36% total N was applied to the first 27 pots before transplanting (^15^N-labeled urea as base fertilizer), 24% total N was applied to additional 27 pots at the mid-tillering stage (nine-leaf stage; ^15^N-labeled urea as tillering fertilizer), and 40% total N was applied to the last 27 pots at the early jointing stage (12-leaf stage; ^15^N-ammonium sulfate as panicle fertilizer). In order to ensure that the total amount of N fertilizer per pot is consistent with the fertilization period, unlabeled N fertilizer is applied except for isotope application. ^15^N-labeled urea (10.18 atom% ^15^N abundance) and ^15^N-ammonium sulfate (10.16% atom% ^15^N abundance) were purchased from Shanghai Research Institute of Chemical Industry in Shanghai, China. In addition, all treatments received the same amounts of phosphorus (180 kg P_2_O_5_ ha^−1^) and potash (120 kg K_2_O ha^−1^) fertilizers. These were dissolved in water and applied before transplanting. All pots were watered regularly to maintain flooded conditions, with the water level 5 cm above the soil surface (except for aeration at the top tillering stage to control effective tillering).

### 2.3 Greenhouse gas emission measurements

The closed static chamber chromatography method was used to monitor N_2_O and methane (CH_4_) gases. Each chamber was 32 cm in diameter × 70 or 120 cm in height, matching the rice growth. The frequency of gas sampling was measured approximately every 2 weeks between 9:00 and 11:00 a.m., with more intensive sampling following fertilization or drainage. The groove was water-filled to ensure good sealing, and the headspace samples were collected at 0-, 15-, 30-, and 45-min intervals after closure and then stored in a 50-mL air-tight syringe. The samples were measured within 3 days as described in Gao et al. (2019) using a gas chromatograph (Agilent 7890A, Agilent Technologies, Santa Clara, CA, USA), and hourly emissions of N_2_O and CH_4_ were determined from the slope of the gas concentration change with four sequential samples. The N_2_O/CH_4_ flux (F, μg N_2_O-N kg^−1^ h^−1^; μg CH_4_-C kg^−1^ h^−1^) was estimated with a linear fit of the sampling time and concentration (Ma et al., 2023) and calculated as Equation 1:


(1) $$F=\rho \times \frac{V}{W}\times \frac{dc}{dt}\times \frac{273}{273+T}$$

where *ρ* (mg cm^−3^) is the standardized state gas density of N_2_O or CH_4_; *V* and *W* represent the volume of the static chamber (m^3^) and dry soil weight of pot (kg), respectively; *dc*/*dt* is the change rate of the N_2_O or CH_4_ concentration in the static chamber (μmol mol^−1^ h^−1^); and *T* is the average temperature of static chamber temperature (°C). The cumulative gas emission (*C*) (mg kg^−1^) was performed in detail as Equation 2:


(2) $$C=\sum_{i=1}^{d}\frac{F_{i+1}+F_{i}}{2}\times (d_{i+1}-d_{i})\times 24$$

where *d* is the sampling date, *i* is the sampling frequency, and 24 is the hours in 1 day.

The N_2_O and CH_4_ emission rates (mg kg^−1^ day^−1^) were divided into three periods, namely, tillering stage (regrowth—late tillering stage, duration 13 days), panicle stage (jointing—booting stage, duration 31 days), and ripening stage (heading—maturity stage, duration 47 days), to evaluate N_2_O emission per unit time or CH_4_ emission per unit time and were calculated as Equation 3 (Gao et al., 2023):


(3) $$N_{2}O\;(CH_{4}\text{ emission rate})\;=\;C/D$$

*C* is the cumulative N_2_O or CH_4_ emissions of rice at three different growth stages (mg kg^−1^), and *D* is the days of rice at three different growth stages (days).

Global warming potential (*GWP*) represents the CO_2_-equivalent impact of CH_4_ and N_2_O emissions (mg CO_2_-eq kg^−1^), which is produced by N_2_O and CH_4_ emissions obtained at a 100-year time horizon using Equation 4:


(4) $$GWP=265\times C_{N_{2}O}+28\times C_{CH_{4}}\;\;$$

where 265 is a conversion factor of N_2_O relative to CO_2_, and 28 is the conversion factor of CH_4_ relative to CO_2_ (IPCC, 2014).

The greenhouse gas intensity (*GHGI*) (mg CO_2_ eq. g^−1^ grain) was used to evaluate GHG emissions per unit rice yield and was calculated as Equation 5 (Maucieri et al., 2017):


(5) GHGI=GWP/Y

where Y is the rice yield (g pot^−1^).

### 2.4 ^15^N fertilizer fate

After rice maturity, two rice plants from each pot were harvested. Plant samples were separated into grains and straw and then oven-dried to a constant weight at 70°C for 48 h and weighed to determine total yield. Grain moisture was determined using a hand-held moisture tester after drying (John Deere, Moline, IL, USA), and grain yield was estimated with a 14.5% moisture content. Dry plant samples were ground and sieved (0.15 mm) to analyze total N content and ^15^N atom% by stable isotope ratio mass spectrometry (DELTA Plus XP, USA). Plant N uptake or soil total N (*TN*), rice organ N accumulation derived from ^15^N-labeled fertilizer (*Ndff*, mg pot^−1^), residual soil ^15^N (*Ndfs*, mg pot^−1^) (Wang et al., 2023), N recovery efficiency (*NRE*, %) (Peng et al., 2017), and the overall contribution rate (*ENR*, %) were calculated as Equations 6–10:


(6) $$TN_{plant}=\text{N accumulated in dry biomass }\times \text{weight of dry biomass}$$

(7) $$Ndff\;({\text{mg pot}}^{-1})=\frac{Af\%-Acf\%}{Au\%-Acf\%}\times TN_{plant\;}\;$$

(8) $$Ndfs\;\;({\text{mg pot}}^{-1})=\frac{Af\%-Acf\%}{Au\%-Acf\%}\times TN_{soil\;}$$

(9) $$NRE(\%)=\frac{N_{ut}-N_{u0}}{N_{applied\;rate}}\times 100\;$$

(10) $$ENR(\%)=\frac{Ndff_{each\;fertiliztion\;period}}{N_{applied\;rate}}\times 100\;\;\;\;\;\;\;$$

where *TN* is the total N content in the plant or soil (mg pot^−1^), and *Au*, *Acf*, and *Af* are the ^15^N abundance in the ^15^N-labeled urea fertilizer (10.18 atom%), natural ^15^N abundance in the plant or soil, and total ^15^N abundance in the plant or soil, respectively. *N_ut_* and *N_u0_* are the N uptake in N treatment and N uptake in N0 treatment, respectively.

^15^N loss rate (NLR, %) and soil residual ^15^N rate in post-harvest (RSE, %) were calculated as Equations 11 and 12:


(11) $$\text{NLR}(\%)=\frac{N_{applied\;rate}-Ndff-Ndfs}{N_{applied\;rate}}\times 100$$

(12) $$\text{RSE}(\%)=\frac{Ndfs}{N_{applied\;rate}}\times 100$$

### 2.5 Statistical analysis

Statistical analyses were performed using SPSS software (version 23.0, SPSS Inc., USA). The particularities of the evaluations required different approaches for analyzing the data. One-way analysis of variance (ANOVA) was used to compare the differences in N_2_O, CH4, and GWP rates across the tillering, panicle, and ripening stages under C0, C1, and C2 treatments, respectively. The one-way ANOVA or the Kruskal–Wallis test was used to examine the differences between treatments for normal and non-normal indicators, respectively. A two-way ANOVA was performed to determine the differences in cumulative N_2_O emissions, cumulative CH_4_ emissions, GWP, NLR, RSE, and NRE between treatments and their interactions, followed by Duncan’s test (*P* < 0.05). When means and standard errors (SE) were reported in this study, the standard deviations (SD) were calculated as: SD = SE $\sqrt{\text{n}}$ , where *n* is the number of replicates. A three-way ANOVA was performed for yield and GHGI, where biochar (C) and nitrogen (N) were considered fixed effects, while year (Y) was considered a random effect. Additionally, the plotting of graphs was conducted using Origin (2021, USA), enabling a precise visualization of the data.

## 3 Results

### 3.1 N_2_O and CH_4_ emissions

N_2_O fluxes varied from −0.57 to 2.37 µg kg^−1^ h^−1^, with an average of 0.46 µg kg^−1^ h^−1^ during the first rice growing seasons (**Supplementary Figure 1**). CH_4_ fluxes ranged from −1.92 to 1.65 μg kg^−1^ h^−1^, with a mean value of 0.50 μg kg^−1^ h^−1^ during the same period. Similar dynamics in N_2_O and CH_4_ fluxes were observed across all treatments in year 2. N_2_O and CH_4_ fluxes in all treatments were recorded, with a mean of 0.13 and 0.034 µg kg^−1^ h^−1^, respectively.

**Figure 2:**
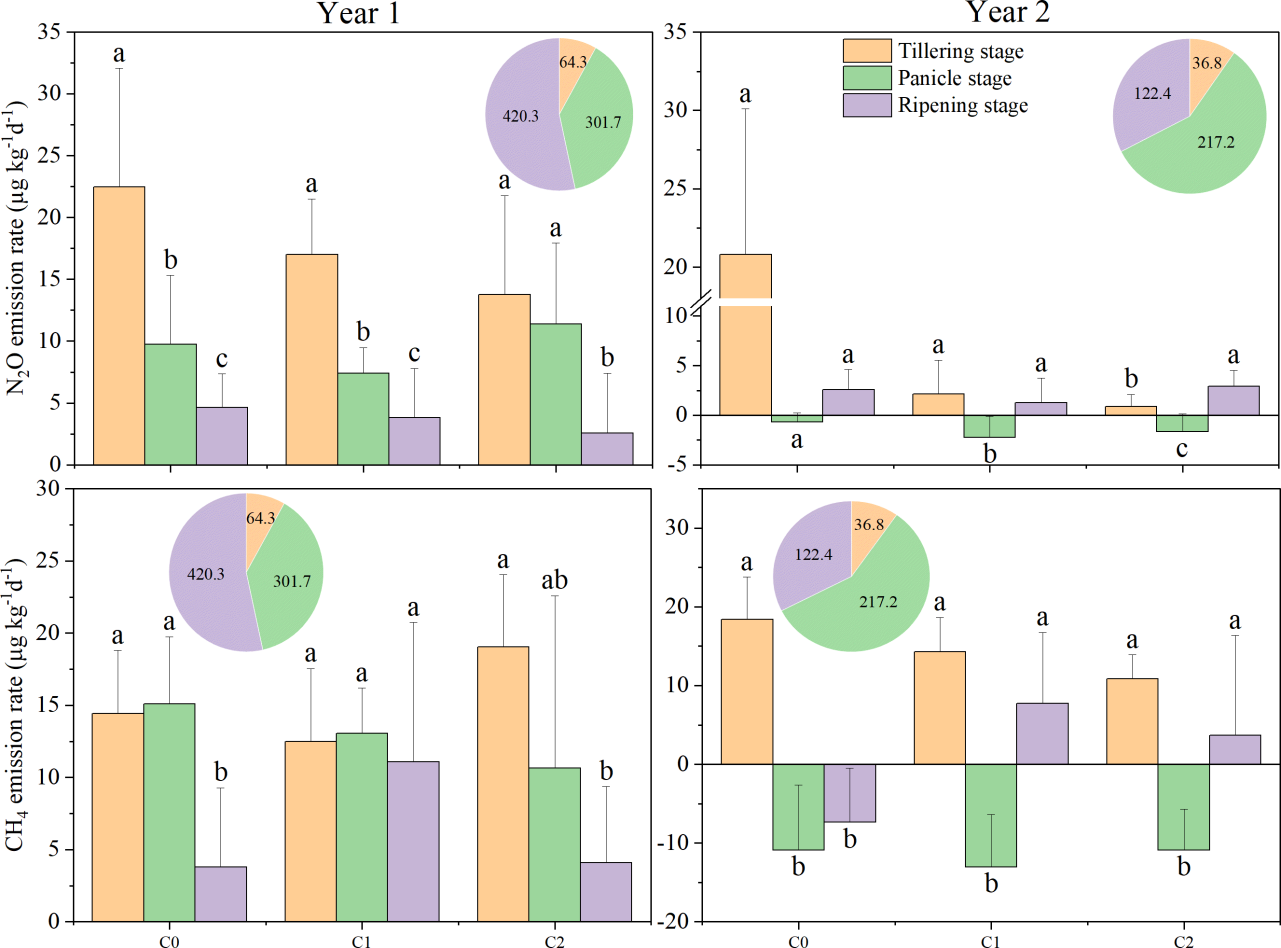
Nitrous oxide (N_2_O) and methane (CH_4_) emission rates in different treatments at various rice growth stages from 2016 to 2017. C0, no biochar application; C1, 15 t ha^−1^ biochar application; C2, 45 t ha^−1^ biochar application. The pie chart represents precipitation at various stages. Different lowercase letters indicate significant differences at *P* < 0.05. The vertical bars represent the standard deviation of the means (*n* = 3).

**Figure 3:**
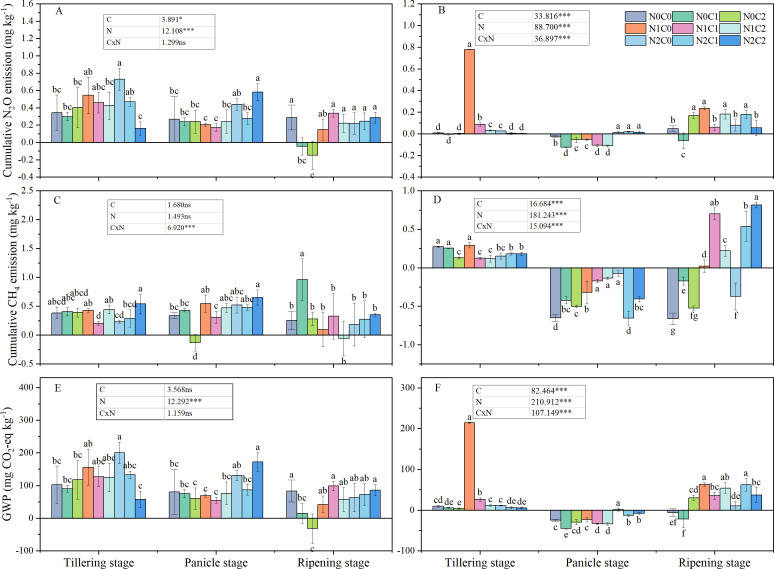
Cumulative N_2_O and CH_4_ emissions and GWP in different treatments at various rice growth stages from 2016 (**A, C, E**) to 2017 (**B, D, F**). N0C0, no biochar and nitrogen addition; N0C1, only 15 t ha^−1^ biochar addition; N0C2, only 45 t ha^−1^ biochar addition; N1C0, only 168 kg N ha^−1^ addition; N1C1, 15 t ha^−1^ biochar addition with 168 kg N ha^−1^; N1C2, 45 t ha^−1^ biochar addition with 168 kg N ha^−1^; N2C0, only 210 kg N ha^−1^ addition; N2C1, 15 t ha^−1^ biochar addition with 210 kg N ha^−1^; and N2C2, 45 t ha^−1^ biochar addition with 210 kg N ha^−1^. Different lowercase letters indicate significant differences at *P* < 0.05. ***P < 0.001; *,0.01 ≤ *P* < 0.05. ns means not significant. Vertical bars represent the standard deviation of the mean (*n* = 3).

N_2_O emission rates in 2017 were lower than in 2016. During growth stages under biochar treatments in 2016, the trend was tillering stage > panicle initiation stage > ripening period (**Figure 2**). This trend was consistent with our assumption that by alternating dry–wet of paddy field, aging accelerates, which alters the surface morphology and physicochemical properties of biochar and induces changes in microbial activity and soil moisture (Luo et al., 2025). In year 2, a minimum N_2_O emission rate (−2.21 to −0.69 µg kg^−1^ day^−1^) occurred during the panicle stage. During tillering of both years, N_2_O emission rates showed a trend of C2 < C1 < C0. During the panicle stage, C1 treatment showed the lowest N_2_O emission rates in both years. During ripening, among different treatments, N_2_O emission rates differed between the 2 years. In year 1, N_2_O emission rates decreased with the increment of biochar; however, the trend of C1 treatment was less 52.3% than C0, which was less 57.9% than C2 in year 2. Cumulative N_2_O emissions exhibited significant variability across different years. In 2016, the cumulative N_2_O emissions for N2C2 were significantly reduced by 53.0% during the tillering stage, but significantly increased by 117.9% during the panicle growth stage, and showed no difference during the ripening period, compared to N0C0 (**Figure 3**). Biochar (C) and nitrogen (N) had a significant impact on cumulative N_2_O emissions; however, the interaction between C and N was significant only in 2017 (**Figure 3**). This inconsistency may depend on different rainfall and soil temperatures in different years.

The CH_4_ emission rate was observed to decline in 2017 compared to that in 2016, with an average reduction of 87.7%. On average, in all treatments, the CH_4_ emission rate of the panicle stage in 2017 significantly decreased by 189.7% compared to 2016, while during the tillering stage, the least reduction of 5.3% was noticed. Throughout the various growth stages with different biochar treatments, a consistent trend was observed: tillering stage > ripening period > panicle growth stage, during the year 2017 (**Figure 2**). In the tillering stage, an increase in biochar correlates with a decrease in CH_4_ emission rates. In the tillering stage, in 2017, cumulative CH_4_ emissions for N0C2, N1C1, and N1C2 were significantly reduced by 53.3%, 54.3%, and 57.3%, respectively, compared to N0C0 (**Figure 3**). Biochar (C) and nitrogen (N) did not significantly affect cumulative CH_4_ emissions in 2016; however, the interaction between C and N was significant in both 2016 and 2017 (**Figure 3**).

### 3.2 Global warming potential

**Figure 4:**
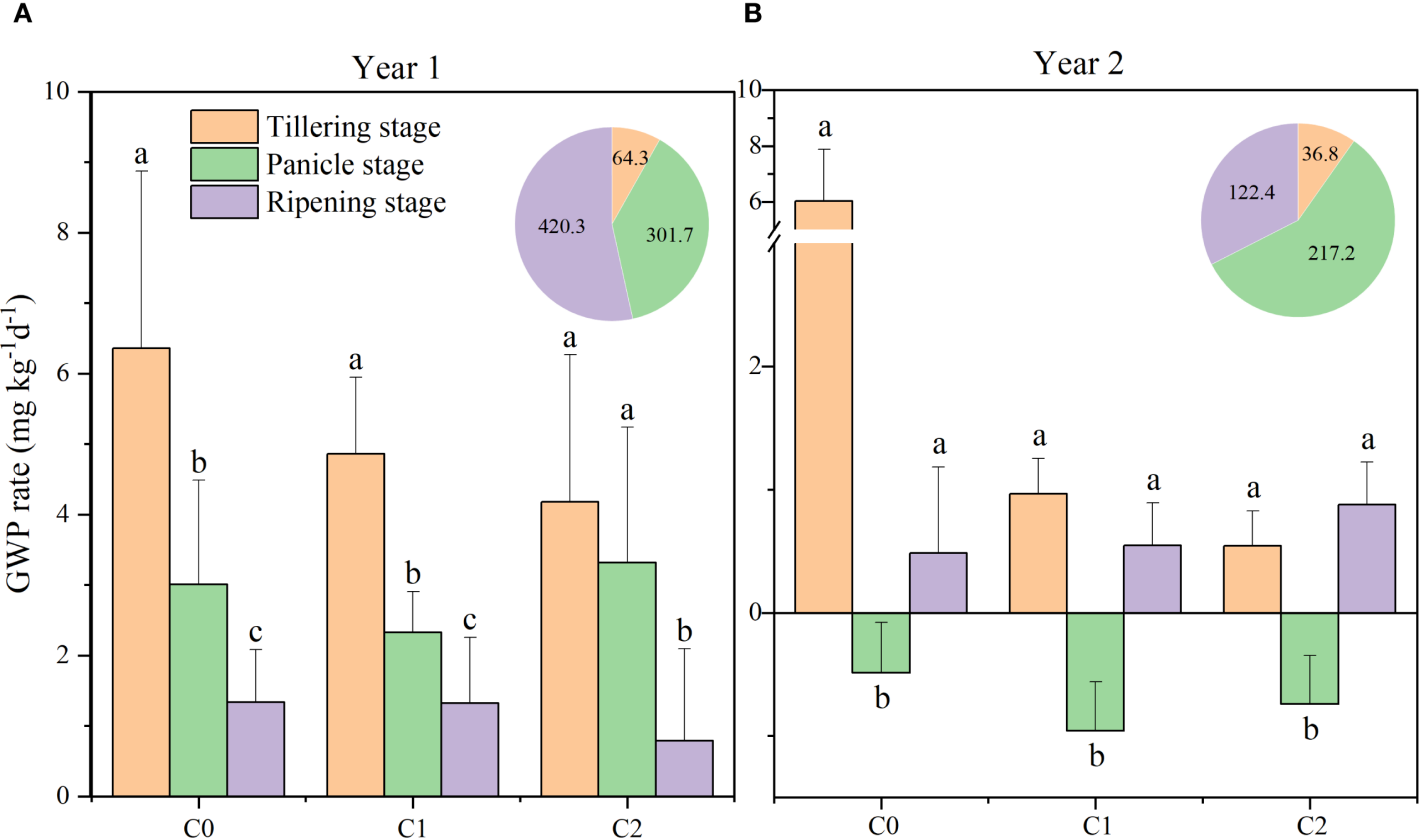
Global warming potential (GWP) rate in different treatments at various rice growth stages from 2016 **(A)** to 2017 **(B)**. C0, no biochar application; C1, 15 t ha^−1^ biochar application; C2, 45 t ha^−1^ biochar application. The pie chart represents precipitation at various stages. Different lowercase letters indicate significant differences at *P* < 0.05. The vertical bars represent the standard deviation of the means (*n* = 3).

In 2016, the average GWP rates across various growth stages were as follows: tillering stage (4.18–6.36 mg kg^−1^ day^−1^) > panicle growth stage (2.33–3.32 mg kg^−1^ day^−1^) > ripening period (0.79–1.34 mg kg^−1^ day^−1^). In 2017, the GWP rate was 0.97–1.62 mg kg^−1^ day^−1^ lower during the panicle growth stage compared to the ripening period (**Figure 4**).

Significant variations in GWP rates were observed across different years (**Supplementary Table 1**). In 2016, biochar application significantly affected the GWP rate only under C2 treatment compared with C0; however, a significant reduction by 28.0% and 25.1% under C1 and C2 treatments was observed in 2017, compared with C0. Compared to the N0C0 treatment, the N2C0 treatment demonstrated GWP by 48.3% (*P* < 0.05), whereas the N0C2 treatment resulted in a 45.0% reduction (*P* < 0.05). No significant difference was observed between the N1C2 and N2C2 treatments. The combined application of biochar and nitrogen resulted in a significant reduction of GWP, with a 44.1% decrease (*P* < 0.05) observed in N1C1 during the ripening period, relative to N1C0. Biochar (C), nitrogen (N), and the interaction of biochar and nitrogen (C × N) significantly influenced the GWP in 2017 (**Figure 3**).

### 3.3 Yield and greenhouse gas intensity

Biochar (C), nitrogen (N), and the interaction of biochar and nitrogen (C × N) significantly influenced rice yield during the 2016–2017 period (**Table 3**). In 2016, the N2C0 treatment significantly enhanced rice yield by 13.0%, compared to N1C0; in the following year, the N2C2 treatment significantly enhanced rice yield by 8.5%. The rice yield increased with the increment of N applications across the 2 years. However, it decreased with biochar additions in 2016, while it initially decreased and subsequently increased in 2017. In 2017, biochar (C), nitrogen (N), and their interaction significantly influenced the GHGI. GHGI was significantly reduced by 66.7%–150.0% in the C1 and C2 treatments compared to the C0 treatment. During 2016, the GHGI of the N2 treatment was decreased by 56.6%, compared to N0. However, the N1 and N2 treatments did not have significant differences. The interaction between nitrogen and biochar became evident solely during the year 2017. At the N1 level, the GHGI of the C1 treatment was recorded at 9.66 mg CO_2_ eq. g^−1^ grain, whereas at the N2 level, the GHGI of the C1 treatment reached 19.61 mg CO_2_ eq. g^−1^ grain.

### 3.4 Fertilizer ^15^N fate

In 2016, the Ndff of basic, tiller, and spike fertilizers decreased at the N1 and N2 levels with increased biochar application. Ndff in basic fertilizer varied from 113.63 to 253.82 mg pot^−1^ (**Supplementary Table 1**), subsequently decreasing to a range of 113.80–145.88 mg pot^−1^. In tiller fertilizer, the highest Ndff values were observed in the N2C1and N2C2 treatments during 2016 and 2017, respectively, resulting in increases of 15.3% and 14.5% compared to N1C0. The recovery of ^15^N from panicle fertilizer was the highest, ranging from 495.37 to 752.88 mg pot^−1^ in 2016. Biochar (C) and nitrogen (N) significantly influenced the Ndff in basic, tiller, and spike fertilizers in 2016; however, the interaction between biochar and nitrogen (C × N) did not show a significant difference (**Supplementary Table 2**). The highest ^15^N recovery amount in the whole growth period was observed in the N2C0 and N2C2 treatments during 2016 and 2017, respectively. The 2-year total ^15^N recovery amount in the whole growth period of N2C2 was increased by 9.6% compared to N2C0 (**Table 2**).

**Table 2:** ^15^N recovery amount in the whole growth period in different treatments.

Year	N1C0	N1C1	N1C2	N2C0	N2C1	N2C2
2016	1,366.29 ± 73.14b	1,319.28 ± 54.57b	1,336.87 ± 252.66b	1,706.43 ± 296.76a	1,553.97 ± 81.95ab	1,581.46 ± 48.43ab
2017	902.30 ± 49.08b	965.67 ± 139.26b	1,179.27 ± 125.25b	1,135.69 ± 95.98b	1,225.19 ± 250.41ab	1,533.21 ± 199.71a

Different lowercase letters indicate significant differences (*P* < 0.05) in the treatments.

The application of biochar at the N1 level in 2016, regardless of whether it was in base, tiller, or spike fertilizer, resulted in a gradual increase in the residual quantity of fertilizer ^15^N (Ndfs). However, Ndfs initially decreased and subsequently increased in base and tiller fertilizers at the N2 treatment. The residual amount of spike fertilizer ^15^N in the N2C2 treatment was significantly increased by 346.0% and 146.2% compared to the N1C0 and N2C0 treatments, with a range of 70.61–314.94 mg pot^−1^ (*P* < 0.05). In comparison to N1C0 and N2C0 in panicle fertilizer, the residual changes in fertilizer ^15^N in 2017 were comparable to those in 2016 and were also the most significant in the N2C2 treatment, increasing by 120.8% and 209.9%, respectively (*P* < 0.05). The tiller fertilizer ^15^N residual soil was the least from the 2-year data. In 2016, the NRS was significantly influenced by biochar (C) and biochar × nitrogen (C × N) in basic, tiller, and spike fertilizers. However, nitrogen (N) did not have a significant effect. Nevertheless, the NRS was significantly impacted by biochar in only the spike fertilizer in 2017 (**Supplementary Table 2**).

The total accumulation of fertilizer ^15^N in the rice–soil system (NRS) for each treatment ranged from 185.40 to 943.92 mg pot^−1^ in 2016. The accumulation of NRS in base, tiller, or spike fertilizer in 2016 was higher than in 2017 (**Supplementary Table 1**). The average total accumulation of fertilizer ^15^N in the rice–soil system for base, tiller, and spike fertilizers was 422.39, 274.04, and 781.08 mg pot^–1^ in 2016 and 351.23, 180.14, and 625.51 mg pot^−1^ in 2017, respectively. The accumulation of ^15^N fertilizer from the tiller fertilizer was lower than that from the base and spike fertilizers. The ENR, similar to Ndff, exhibited varying averages across different fertilization periods over 2 years, ranked as follows: spike fertilizer (23.57%–32.00%) > base fertilizer (5.41%–12.09%) > tiller fertilizer (6.58%–11.75%). Notably, the average ENR in 2017 was lower than that in 2016.

**Figure 5:**
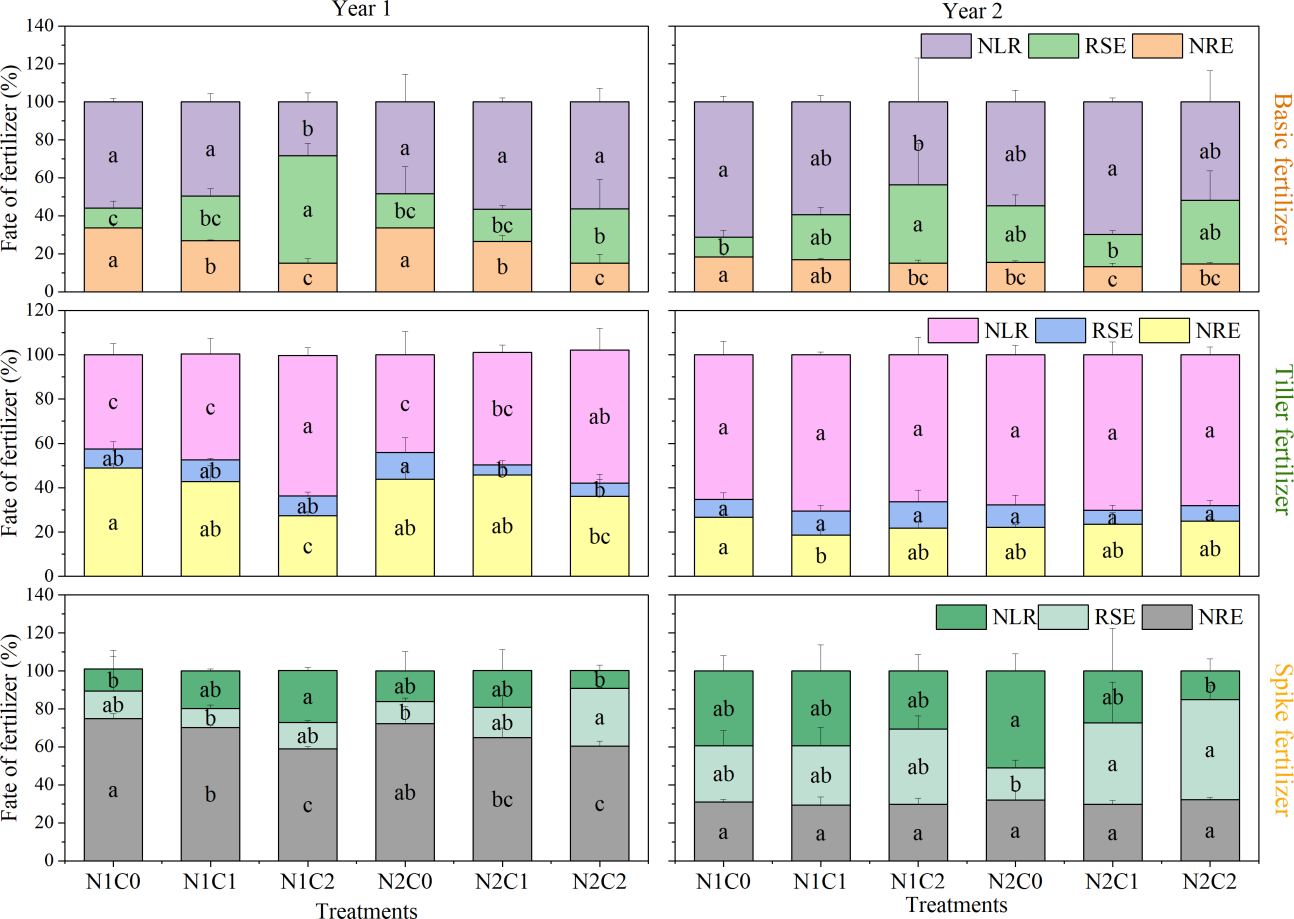
^15^N loss rate (NLR), soil residual ^15^N rate in post-harvest (RSE), and N recovery efficiency (NRE) in different treatments from 2016 to 2017. N1C0, only 168 kg N ha^−1^ addition; N1C1, 15 t ha^−1^ biochar addition with 168 kg N ha^−1^; N1C2, 45 t ha^−1^ biochar addition with 168 kg N ha^−1^; N2C0, only 210 kg N ha^−1^ addition; N2C1, 15 t ha^−1^ biochar addition with 210 kg N ha^−1^; and N2C2, 45 t ha^−1^ biochar addition with 210 kg N ha^−1^. Different lowercase letters indicate significant differences (*P* < 0.05) in the treatments. The vertical bars represent the standard deviation of the means (*n* = 3).

The influence of biochar on ^15^N loss rate (NLR) varied across different fertilization periods (**Figure 5**). The N1C2 treatment resulted in a significant reduction of the NLR by 49.5% and 38.6% for the base fertilizers of 2016 and 2017, respectively, when compared to N1C0 (*P* < 0.05; **Figure 5**). The N1C2 treatment in the tiller or spike fertilizer resulted in a significant increase in NLR by 49.2% and 135.2% compared to N1C0 in 2016, respectively. Furthermore, regarding the NRE in base, tiller, or spike fertilizer in 2016, the N1C2 treatment exhibited lower values of 55.2%, 44.0%, and 21.4%, compared to N1C0 (*P* < 0.05; **Figure 5**). However, compared with N1C0, the NRE of base fertilizer under N2C1 experienced a significant decrease by 27.9% in 2017. Biochar had no significant effect on the NRE of tillering and ear fertilizer in 2017.

## 4 Discussion

### 4.1 N_2_O emissions and residual soil ^15^N

Our results showed that the N_2_O emission rates in the tilling and ripening stages under biochar treatments were higher in year 1 than in year 2, which could be attributed to significant changes in soil characteristics in the initial year (**Figure 2**). The interannual variations may be related to the aging of biochar. The reduction of N_2_O emission rates in 2017 can be attributed to two primary factors. First, aging biochar primarily affects soil GHG emissions in the short term by reducing biochar dissolved organic carbon (DOC) release and promoting the humification of biochar dissolved organic matter (DOM) (Pan et al., 2024). Finally, aging biochar promotes microbial nitrogen fixation (Zhou et al., 2024). On one hand, the aging of biochar can lead to a reduction in specific surface area, thereby facilitating the absorption of soil organic pollutants. This process impedes the survival and activity of microorganisms within the biochar pores (Yang et al., 2022). Conversely, biochar aging can also result in an acidification effect, which may adversely affect soil phosphorus bioavailability and subsequently reduce microbial activity (Zhang et al., 2025b; Wang et al., 2020a). Meanwhile, we observed a clear seasonal effect of 45 t ha^−1^ biochar combined with N treatment in year 1 (**Figure 3**), with lower cumulative N_2_O emissions during the tillering stage, which is similar to the results of Shen et al. (2024). The same pattern was observed in the second year, indicating that the interaction may have depended in part on crops needing fertilizer. It is too early for our study to fully account for the relationship between biochar aging and the split application of nitrogen fertilizers. Previous studies indicated that medium (10 t ha^−1^) and high (20 t ha^−1^) amounts of biochar have different potential to influence N_2_O emissions (Li et al., 2022b; Zhang et al., 2021), which is similar to our study. This suggests that biochar reduced the emissions of paddy fields by affecting the N_2_O emission rate. Our study demonstrates that N_2_O and CH_4_ emissions in rice at different periods can be related to precipitation, which means that larger amounts of N_2_O emissions that evolved from paddy soils are related to soil wetting–drying cycles (Verhoeven et al., 2018). Biochar applied in the first year, which induced short-term priming effects on the N_2_O emission rate or cumulative emissions that occurred in the different rice stages, was approximately higher at 38.6%–115.9% or 39.9–116.4% than that in the second year, which is consistent with Wu et al. (2021), who observed that high N_2_O emissions that occurred in the BC10 treatment (chemical fertilizer combined with 10 t ha^−1^ biochar) showed a declining trend at 16.4%–18.0% during the next 2 years. Meanwhile, in our study, during the panicle stage and ripening stage in 2016, N_2_O emissions under the N2C2 treatment were higher than in the N2C0 treatment by 17.3% and 49.9%, respectively. However, in the next year, the N2C2 treatment showed lower N_2_O emissions than the N2C0 treatment. Our findings were consistent with prior research showing that biochar had little effect in reducing N_2_O emissions (Verhoeven and Six, 2014). However, there was no significant variation between the N2C2 and N2C0 treatments in 2017, which is in contrast with Xu et al. (2014), who observed that low N_2_O emissions occurred after biochar addition to the pot trial. This inconsistency may depend on low-temperature pyrolysis, and biochar physicochemical properties showed a slowly changing trend at temperatures above 400°C (Chen et al., 2025). The subsequent season (2017) saw a larger improvement in N_2_O emission, which decreased under high biochar addition levels compared with the first season (2016) (**Figure 3**). This could be due to biochar aging, increasing its adsorption capability, which contributed to the adsorption of inorganic nitrogen (Li et al., 2022a; Wang et al., 2020b). Biochar reduced the N_2_O emission rate, but it did not reduce cumulative N_2_O emissions in the first year of the panicle growth stage and ripening stage. However, biochar reduced N_2_O emissions during the tilling stage, suggesting that biochar’s alkalinity inhibited the nitrification pathway in the early stages of rice and altered the soil pH in the later stages (Islam et al., 2018). In this study, N_2_O emissions showed no significant effect among the interaction between biochar and nitrogen in the first year; however, significant interaction effects were found in the following years. Due to adsorption, the retention capacity of fresh biochar was more robust than the reduction in denitrification (Shi et al., 2024). However, in the second year, biochar maybe inhibit the denitrification process. This change indicated that nitrogen with biochar may influence the soil carbon utilization rate’s microbial community, but it would not have a major impact right away. This will alter N_2_O emissions and encourage denitrification in the second year (Vilain et al., 2014).

We found that biochar and N interaction significantly influenced CH_4_ emissions in the two rice growing seasons, and the interaction becomes stronger in the second year. Biochar increased the CH_4_ emission rate only in the second year of the ripening stage, which is consistent with the findings of Li et al. (2025) and Wu et al. (2019). This accelerative effect can improve the electron transfer capacity of extracellular polymeric substances (EPS), which subsequently enriches electroactive microorganisms resulting from biochar-induced N-acyl homoserine lactones (AHLs) (Li et al., 2025). Another reason is potentially due to a decrease in soil pH induced by aged biochar addition (Wu et al., 2019). Conversely, aged biochar at a low addition ratio (1%) decreased CH_4_ emission, potentially attributed to enhancing soil aeration and promoting methane-oxidizing bacteria (Kubaczyński et al., 2022).

According to earlier research, the risks for N drainage from paddy fields occur 7 days after basal fertilization and 5 days after tillering fertilization (Zhang et al., 2019b). Based on an experiment investigating the efficiency of nitrogen fertilizer during various rice growth periods, biochar has certain agricultural advantages in the transformation and utilization of N fertilizer (Zhang et al., 2022). Our results showed that low nitrogen combined with high-dose biochar treatment (N1C2) promoted soil residual ^15^N of basic fertilizer applied to rice during 2016–2017, showing an average increase of 46.3% and 30.8% compared to the low nitrogen only treatment (N1C0). This result is similar to Ning et al. (2022), indicating that biochar enhanced the residual rate of nitrogen fertilizer in cornfields. In addition, we found that soil residual ^15^N from tiller fertilizer showed no significant effect under biochar treatments (**Figure 5**). In contrast, conventional nitrogen combined with the high-dose biochar treatment (N2C2) has the greatest impact on soil residual ^15^N in spike fertilizer, comprising 18.9%–35.7% of the total spike fertilizer. This effect was most pronounced in the second year following biochar application, aligning with the findings from prior studies (Qian et al., 2023; Hossain et al., 2020). This may be attributed to i) the long-term function of biochar as a slow-release nitrogen agent, which facilitates the transformation of residual nitrogen fertilizer in the surface soil, particularly enhancing the formation of nitrates in the soil solution (Shi et al., 2022; Yan et al., 2018); and ii) the increased levels of biochar, which possesses higher adsorption capacity and water retention ability, thereby augmenting the prevention of nutrient loss and improving nutrient supply (Jin et al., 2019). The complex aromatic structure of biochar may contribute to reduced nitrogen release in the soil (Lehmann et al., 2021). Subsequent studies indicated that biochar markedly enhanced residual nitrogen in the topsoil, while it diminished the nitrogen transformation efficiency in the soil layer below 20 cm (Shi et al., 2024). This study indicated that biochar affected the soil nitrogen residue from base and panicle fertilizers, but did not have a significant impact on tillering fertilizer (**Figure 5**). This lack of impact may be attributed to the primary role of tillering fertilizer in promoting tillering (Zhang et al., 2024a), coupled with the requirement to dry the rice field at the conclusion of the tillering phase, which subsequently leads to a surge in N_2_O emissions (Xu, 2021). The impact of intermittent irrigation during the booting and flowering stages was more significant than that of biochar on tillering fertilizer, leading to no discernible impact on tillering fertilizer in this study.

### 4.2 ^15^N recovery rates and grain yield

NRE serves as a crucial indicator of nitrogen fertilizer absorption and utilization during plant growth. The pathways to enhance NRE have been the subject of extensive research (Sun et al., 2019). Biochar-mediated processes vary across different stages of plant growth. The increase in Ndff observed in the N2C1 and N2C2 treatments might be attributable to decreased N_2_O emissions during the panicle and ripening stages (**Figure 3**). The aging of biochar leads to higher levels of oxygen-containing functional groups (Singh et al., 2022) and the lowering of the denitrification substrate (Zhou et al., 2024), and this may be the reason for the lower Ndff in 2017 than in 2016. Previous studies revealed that biochar influences the sorption of NH_4_^+^ and NO_3_^−^ ions in the soil, while moderated soil aeration enhances nitrifier activity, thus improving nitrification and NUE (Khan et al., 2023; Gao et al., 2022). In two rice seasons, the N1C2 treatment increased the soil residual ^15^N rate (RSE) of basic fertilizer, compared to the other treatments, and the N2C2 treatment increased the RSE of spike fertilizer. This might be because biochar’s adsorption and retention of fertilizers is stronger than the microorganisms’ decomposition in short-term experiments. The increase in soil residual ^15^N was attributed to the adsorption of N by biochar due to its porous structure and functional groups (Chen et al., 2021). In this study, we revealed that biochar markedly decreased the NRE of base fertilizer and spike fertilizer, with this effect diminishing in the second year (**Supplementary Table 3**), consistent with the results of Gaskin et al. (2010). Previous studies indicated that biochar improved NUE in soils with low pH and organic matter content in southern China, while its effectiveness diminishes in northeast China, which is characterized by high organic matter content and pH (Xia et al., 2021). This study found that soil capacity in pot experiments is limited relative to field experiments, likely due to excessive biochar that limited crop root development, especially the lateral root, which affects N uptake (Li et al., 2022a; Xia et al., 2022). Meanwhile, we demonstrated that biochar reduced N_2_O emissions during the tillering stage and ripening stage. This inhibitory effect can be attributed to the microaerobic conditions created by biochar in the tillering stage to inhibit denitrification, ensuring N_2_O is converted to N_2_. Biochar increased soil moisture content. Although the drainage environment during the ripening stage is beneficial, its moisture content is still higher than that without biochar addition, which is not conducive to denitrification and prevents N_2_O emission (Cayuela et al., 2014). Furthermore, it did not improve the NRE of base or spike fertilizers; however, biochar increased the residual soil N. These studies show that the impact of biochar on crop NRE is primarily associated with soil organic matter content and biochar type (Van Zwieten et al., 2010). However, the specific mechanism of high soil organic matter content inhibiting the absorption and utilization of soil inorganic N by plants is not clear and needs further investigation. Future research could improve our knowledge on the influence of biochar on NRE during fertilization at different growth stages of rice, by investigating the potential interrelations among these factors.

The present study found that N fertilizer had a more significant impact on rice yield compared to biochar (**Table 3**). No notable variances in rice yields were observed under the only biochar addition treatments in the first year. We found annual variations in rice grain yield and significant differences among the biochar-combined N treatments (*P* < 0.05). Qin et al. (2016) also observed similar interannual variations. At the same N level, the rice yield was higher under low-dose biochar (15 t ha^−1^) than high-dose biochar (45 t ha^−1^); however, the opposite result occurred during the following year. Additionally, we found that the effect of biochar on rice yield was more pronounced under high N fertilization (N2) than under low N fertilization (N1) conditions (**Table 3**). Similar results were reported by Spokas et al. (2012), with approximately half of the biochar studies showing negative effects on yield in the short term. These results can be attributed to the fact that adding biochar with a high C/N ratio to soil might lead to N loss in the tiller fertilizer and spike fertilizer, affecting the soil profile, and hence, reducing the N uptake in the first year (Qin et al., 2016). However, in the second year, aging biochar resulted in an increase in soil total N and/or rice production (Zhou et al., 2024). However, our results are inconsistent with previous studies (Nguyen et al., 2016; Lai et al., 2013). For example, there is a notable distinction in the short-term experiments (≤5 years), which resulted in yield increasing by 9.1% to 27% (Zhang et al., 2024b; Liu et al., 2019). In contrast, biochar one-off application exceeding 5 years did not influence yield (Zhang et al., 2024b). Field studies indicate that high-dose biochar combined with N increased rice yield, but the increment diminished after 6 years (Liu et al., 2022). The increased crop yield is attributable to management practices and the physical and chemical properties of the soil, which create a more favorable growth environment for crop roots, thereby enhancing crop water productivity (Xiao et al., 2024; Jiang et al., 2024). This phenomenon may be attributed to heterogeneity in biochar production conditions, which is a critical factor contributing to these contrasting findings. Biochar produced from different feedstocks and under varying pyrolysis conditions exhibits distinct properties that can significantly influence the soil nitrogen cycle (Davys et al., 2023). Future research should investigate the residual effects of biochar on the early stage of rice growth and yield formation.

**Table 3:** Rice yield and GHGI under biochar and nitrogen treatments in 2 years.

Treatments		Yield (g pot^−1^)		GHGI (mg CO_2_ eq. g^−1^ grain)	
		2016	2017	2016	2017
N0	C0	22.37 ± 1.68f	28.50 ± 1.51d	0.63 ± 0.03a	−0.05 ± 0.01d
	C1	20.48 ± 1.68f	24.25 ± 1.51e	0.63 ± 0.03a	−0.18 ± 0.01e
	C2	22.51 ± 1.68f	25.43 ± 1.51e	0.47 ± 0.03b	0.01 ± 0.01c
N1	C0	85.74 ± 2.05b	79.53 ± 1.71b	0.22 ± 0.03c	0.23 ± 0.01a
	C1	73.03 ± 1.78d	71.62 ± 1.71c	0.28 ± 0.03c	0.03 ± 0.01bc
	C2	67.52 ± 1.90e	75.38 ± 1.51bc	0.28 ± 0.03c	0.03 ± 0.01bc
N2	C0	101.06 ± 1.90a	86.97 ± 1.85a	0.28 ± 0.03c	0.02 ± 0.01c
	C1	80.12 ± 1.68bc	78.53 ± 1.60b	0.26 ± 0.03c	0.05 ± 0.01b
	C2	74.72 ± 1.90cd	88.34 ± 1.51a	0.29 ± 0.03c	0.03 ± 0.01bc
Nitrogen					
N0		21.79 ± 0.97c	26.06 ± 0.87c	0.58 ± 0.02a	−0.07 ± 0.00c
N1		75.43 ± 1.11b	75.51 ± 0.95b	0.26 ± 0.02b	0.10 ± 0.01a
N2		85.30 ± 1.06a	84.61 ± 0.96a	0.28 ± 0.02b	0.03 ± 0.01b
Biochar					
C0		69.73 ± 1.09a	65.00 ± 0.98a	0.38 ± 0.02a	0.06 ± 0.01a
C1		57.88 ± 0.99b	58.14 ± 0.93b	0.39 ± 0.02a	−0.03 ± 0.00c
C2		54.92 ± 1.06c	63.05 ± 0.87a	0.34 ± 0.02a	0.02 ± 0.00b
ANOVA *P*-values					
Nitrogen (N)		1,549.637***	2,055.594***	103.980***	318.485***
Biochar (C)		41.928***	16.709***	2.113NS	93.787***
N × C		11.606***	1.809NS	4.834**	106.470***
N × C × Y		16.947***		232.058***	

GHGI, greenhouse gas intensity; C0, no biochar application; C1, 15 t ha^−1^ biochar application; C2, 45 t ha^−1^ biochar application. N0, no N application; N1, 168 kg ha^−1^ N application; N2, 210 kg ha^−1^ N application. Different lowercase letters indicate significant differences (*P* < 0.05) among treatments. **, and *** mean significance at the 0.01, and 0.001 levels, respectively. NS means not significant. Values are mean ± SE (*n* = 3). Y represents the year.

## 5 Conclusions

Only biochar addition was more effective in reducing N_2_O emissions at the tillering stage than that combined with N fertilizer. N_2_O emission rates varied across rice growth stages in the first year. Initial biochar application negatively affected nitrogen derived from fertilizer. Spike fertilizer contributed the most to the N recovery efficiency, whereas biochar had varying effects in different fertilization periods. The highest rice yields in 2016 and 2017 were attained using the N2C0 and N2C2 treatments, respectively, which were higher by 13.0% and 8.5% compared with N1C0. This result was based on a short-term pot, and the field conditions were not considered. Therefore, in subsequent studies, biochar and supplemental fertilization should be optimally combined to maximize rice yield while minimizing greenhouse gas emissions, as well as the mechanisms underlying the interaction between biochar and different fertilization periods (base, tillering, and spike) in rice production. Optimal agricultural practices should consider both biochar and supplemental fertilization timing and dosage to balance the yield, greenhouse gas emissions, and NRE. Due to the limitations of pot experiments, the aging period of biochar was shortened. This study recommends that future research should focus on soil residual N processes and microbial functions under different fertilizers in field-based experiments to better support the large-scale practical application of biochar.

## Data Availability

The raw data supporting the conclusions of this article will be made available by the authors, without undue reservation.
