# Supplementary material for: Biochar one-off application for paddy soil 15N loss improvement: evidence from a two-year experiment
Source: Front Plant Sci. 2025 Oct 28;16:1683435. doi: 10.3389/fpls.2025.1683435 (PMC12602487; doi:10.3389/fpls.2025.1683435)
Supplement: Supplementary file 1 [file Table1.docx]

Supplementary Table

**Supplementary Table 1** The average of GWP rate to all treatments across different years.

| Year | Tillering stage | Panicle stage | ripening stage |
| --- | --- | --- | --- |
| 2016 | 5.13±2.12a | 2.88±1.44a | 1.15±1.01a |
| 2017 | 2.51±5.06b | -0.73±0.45b | 0.64±0.67b |

**Supplementary Table 2** Ndff, Ndfs, NRS and ENR in different treatments in 2016-2017.

| **Period** | **Treatments** | **Ndff (****mg pot^−1^)** | |  | **Ndfs (mg pot^−1^)** | | **NRS (mg pot^−1^)** | | **ENR (%)** | |
| --- | --- | --- | --- | --- | --- | --- | --- | --- | --- | --- |
|  |  | **2016** | **2017** |  | **2016** | **2017** | **2016** | **2017** | **2016** | **2017** |
| Basic fertilizer | N1C0 | 253.82±16.87b | 138.97±8.36a |  | 78.19±27.59c | 78.40±28.00b | 332.00±13.70c | 217.37±21.72b | 12.08±0.80a | 6.61±0.40a |
|  | N1C1 | 202.32±4.08c | 127.71±5.87ab |  | 177.97±29.45bc | 178.68±29.14ab | 380.30±33.09bc | 306.39±24.79ab | 9.63±0.19b | 6.08±0.28ab |
|  | N1C2 | 113.63±18.83d | 113.80±12.71b |  | 427.66±48.91a | 311.10±162.66a | 541.29±35.67a | 424.91±174.30a | 5.41±0.90c | 5.41±0.60bc |
|  | N2C0 | 314.71±3.00a | 145.88±6.92a |  | 169.24±134.51bc | 279.02±53.08a | 483.95±135.74ab | 424.90±57.03a | 12.09±0.12a | 5.61±0.27bc |
|  | N2C1 | 248.08±31.50bc | 124.52±17.48ab |  | 159.68±18.06bc | 158.81±18.48ab | 407.76±19.31abc | 283.33±19.11ab | 9.53±1.21b | 4.79±0.67c |
|  | N2C2 | 142.13±42.86d | 137.81±7.35a |  | 246.92±55.73b | 312.69±147.10a | 389.05±66.07bc | 450.50±152.45a | 5.46±1.65c | 5.30±0.28bc |
| Tiller fertilizer | N1C0 | 247.04±10.35ab | 135.35±17.38ab |  | 44.11±16.43b | 40.54±14.55a | 291.15±25.93ab | 175.89±30.46a | 11.75±0.49a | 6.44±0.83a |
|  | N1C1 | 216.33±37.43b | 94.38±9.18c |  | 47.73±2.21ab | 54.76±12.87a | 264.05±35.75b | 149.14±5.92a | 10.29±1.78ab | 4.49±0.44b |
|  | N1C2 | 138.33±9.26c | 110.12±13.96bc |  | 47.07±9.41ab | 60.00±26.69a | 185.40±18.07c | 170.12±39.86a | 6.58±0.44c | 5.24±0.66ab |
|  | N2C0 | 272.53±38.23ab | 137.63±7.42ab |  | 76.07±40.89a | 63.26±26.98a | 348.59±65.65a | 200.89±26.48a | 10.47±1.47ab | 5.29±0.29ab |
|  | N2C1 | 284.83±24.09a | 146.94±32.71a |  | 21.69±3.48b | 39.02±14.37a | 306.52±20.97ab | 185.96±36.14a | 10.95±0.93ab | 5.65±1.26ab |
|  | N2C2 | 224.60±47.83ab | 154.94±13.69a |  | 23.89±14.29b | 43.92±14.57a | 248.49±60.37bc | 198.86±21.59a | 8.63±1.84bc | 5.95±0.53ab |
| Spike fertilizer | N1C0 | 672.52±20.87ab | 260.62±11.67b |  | 70.61±42.25c | 248.43±67.62bc | 743.13±57.53bc | 509.05±67.51b | 32.00±0.99a | 12.40±0.56a |
|  | N1C1 | 589.91±10.28b | 246.73±35.45b |  | 85.02±15.88c | 263.42±79.50bc | 674.93±8.45cd | 510.14±114.87b | 28.07±0.49b | 11.74±1.69a |
|  | N1C2 | 495.37±11.09c | 250.44±28.13b |  | 115.55±9.75bc | 333.80±57.40abc | 610.91±14.73d | 584.24±72.94b | 23.57±0.53c | 11.92±1.34a |
|  | N2C0 | 752.88±93.51a | 332.85±52.79a |  | 121.01±19.61bc | 177.05±42.36c | 873.89±106.37a | 509.90±94.72b | 28.93±3.59ab | 12.79±2.03a |
|  | N2C1 | 675.39±53.16ab | 309.67±22.30ab |  | 164.29±67.22b | 446.23±223.70ab | 839.69±115.84ab | 755.90±233.73ab | 25.96±2.04bc | 11.90±0.86a |
|  | N2C2 | 628.97±26.72b | 335.26±12.14a |  | 314.94±4.57a | 548.58±76.29a | 943.92±29.87a | 883.85±65.91a | 24.17±1.03c | 12.88±0.47a |

Note: Ndff, N in rice plants from ^15^N fertilizer; Ndfs, soil residual ^15^N; NRS, ^15^N in rice-soil system; ENR, the percentage of ^15^N recovery of each period in total N application. Means within each column and year followed by different lowercase letters were significantly different at *P* < 0.05

**Supplementary Table 3** A two-way ANOVA for the effects of biochar (B), nitrogen (N) on Ndff, Ndfs, NRS, ENR, and NRW in 2016-2017. * and * * mean significance at the 0.05 and 0.01 levels, respectively. NS means not significant.

| Stage | Source | Ndff | | Ndsf | | NRS | | NRW | |
| --- | --- | --- | --- | --- | --- | --- | --- | --- | --- |
|  |  | 2016 | 2017 | 2016 | 2017 | 2016 | 2017 | 2016 | 2017 |
| Basic fertilizer | B | 58.992*** | 4.596* | 15.747*** | 3.908NS | 1.694NS | 3.222NS | 0.681NS | 6.519* |
|  | N | 14.399** | 3.258NS | 1.207NS | 1.695NS | 0.073NS | 2.050NS | 13.935** | 12.461** |
|  | NⅹB | 0.621NS | 2.403NS | 5.810* | 2.261NS | 6.970* | 2.059NS | 0.211NS | 0.210NS |
| Tiller fertilizer | B | 11.250** | 1.186NS | 4.793* | 0.136NS | 11.278** | 0.774NS | -- | -- |
|  | N | 16.621** | 14.467** | 0.571NS | 0.111NS | 9.102* | 4.311NS | -- | -- |
|  | NⅹB | 1.499NS | 3.202NS | 6.145* | 1.999NS | 0.117NS | 0.058NS | -- | -- |
| Spike fertilizer | B | 17.293*** | 0.522NS | 18.549*** | 5.789* | 0.897NS | 4.361* | -- | -- |
|  | N | 22.779*** | 22.037*** | 43.099*** | 3.858NS | 44.389*** | 8.570* | -- | -- |
|  | NⅹB | 0.658NS | 0.165NS | 7.455** | 2.674NS | 3.954NS | 2.185NS | -- | -- |

**Supplementary Table 4** A two-way ANOVA for the effects of biochar (B), nitrogen (N) on NRE of basic fertilizer, tiller fertilizer and spike fertilizer in 2016-2017. * and * * mean significance at the 0.05 and 0.01 levels, respectively. NS means not significant.

| Treatments | Basic fertilizer | | Tiller fertilizer | | Spike fertilizer | |
| --- | --- | --- | --- | --- | --- | --- |
|  | 2016 | 2017 | 2016 | 2017 | 2016 | 2017 |
| C0 | 33.61±1.28a | 16.99±0.92a | 46.26±4.09a | 24.41±2.31a | 73.63±5.73a | 31.48±3.23a |
| C1 | 26.64±1.95b | 15.10±1.32b | 44.22±5.63a | 21.11±3.53a | 67.50±3.16b | 29.54±3.18a |
| C2 | 15.11±3.53c | 14.89±1.23b | 31.68±4.75b | 23.31±2.48a | 59.65±1.94c | 30.99±2.26a |
| N1C0 | 33.64±2.24a | 18.42±1.11a | 48.80±2.05a | 26.74±3.43a | 74.98±2.48a | 30.99±1.39a |
| N1C1 | 26.81±0.54b | 16.92±0.78ab | 42.74±7.39ab | 18.64±1.81b | 70.15±1.22b | 29.34±4.22a |
| N1C2 | 15.06±2.50c | 15.08±1.68bc | 27.33±1.83c | 21.75±2.76ab | 58.91±1.32c | 29.78±3.35a |
| N2C0 | 33.58±0.32a | 15.56±0.74bc | 43.72±6.13ab | 22.08±1.19ab | 72.29±8.98ab | 31.96±5.07a |
| N2C1 | 26.47±3.36b | 13.28±1.87c | 45.70±3.86ab | 23.57±5.25ab | 64.85±5.10bc | 29.73±2.14a |
| N2C2 | 15.16±4.57c | 14.70±0.78bc | 36.03±7.67bc | 24.86±2.20ab | 60.39±2.57c | 32.19±1.17a |
| ANOVA *P* values | | | | | | |
| Biochar (C) | 66.701*** | 5.052* | 12.921** | 1.616NS | 21.936*** | 0.504NS |
| Nitrogen (N) | 0.006NS | 14.899** | 0.750NS | 0.544NS | 3.564NS | 0.586NS |
| NⅹC | 0.010NS | 2.742NS | 2.486NS | 3.709NS | 1.827NS | 0.134NS |
