# Supplementary figures and images for: Biochar one-off application for paddy soil 15N loss improvement: evidence from a two-year experiment
Source: Front Plant Sci. 2025 Oct 28;16:1683435. doi: 10.3389/fpls.2025.1683435 (PMC12602487; doi:10.3389/fpls.2025.1683435)

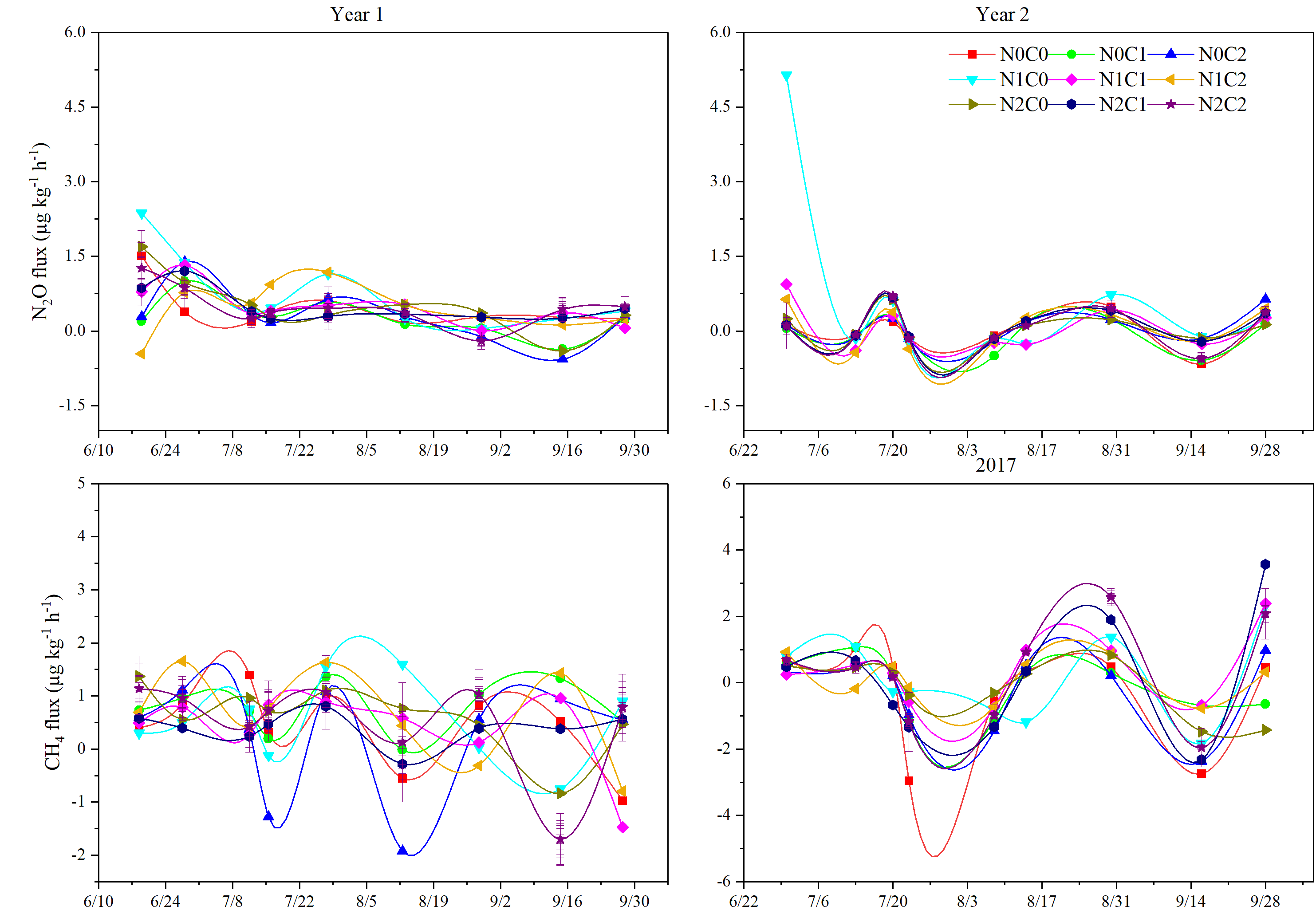

Supplement: Supplementary file 2 [file Image1.tif]
